# The origin of cultivated mangosteen (*Garcinia mangostana* L. var. *mangostana*): Critical assessments and an evolutionary‐ecological perspective

**DOI:** 10.1002/ece3.9792

**Published:** 2023-03-16

**Authors:** T. L. Yao, M. Nazre, D. McKey, R. Jalonen, J. Duminil

**Affiliations:** ^1^ DIADE University of Montpellier, IRD, CIRAD Montpellier France; ^2^ Faculty of Forestry and Environment Universiti Putra Malaysia Serdang Selangor Malaysia; ^3^ Forestry and Environment Division Forest Research Institute Malaysia Kepong Selangor Malaysia; ^4^ CEFE, University of Montpellier, CNRS, EPHE, IRD Montpellier France; ^5^ Alliance of Bioversity International and CIAT Asia – Malaysia Office, c/o WorldFish Headquarters Penang Malaysia

**Keywords:** evolutionary ecology, forest‐*dusun* interface, fruit tree domestication, *Garcinia mangostana*, wild relatives

## Abstract

Mangosteen (*Garcinia mangostana* var. *mangostana*) is a popular tropical fruit, yet many aspects of its biology and evolutionary history are little known. Its origin remains contentious, although recent findings suggest *G*. *mangostana* L. var. *malaccensis* (Hook. *f*.) Nazre (synonym: *G*. *malaccensis* Hook. *f*.) as the sole progenitor. We review hypotheses on the origin of mangosteen and clarify points that have been affected by errors of fact and interpretation. The narrow focus and lack of detail in published results make their interpretation difficult. When possible, we support our interpretations with field observations and examination of herbarium specimens. We outline the main biological traits (e.g., dioecy, facultative apomixis, and polyploidy) of mangosteen and its wild relatives to infer traits that might have evolved during domestication of mangosteen. We find no clear indication that apomixis and polyploidy evolved during domestication. Polyploidy is known in the wild relatives, but apomixis has not yet been demonstrated. Also, we propose a testable new evolutionary‐ecological framework that we call “Forest‐*Dusun* Interface” to infer processes in the origin of mangosteen. *Dusun* (Malay) refers to subsistence orchards in this context. Lastly, we propose future studies to address identified knowledge gaps.

## 1 INTRODUCTION

Mangosteen (*Garcinia mangostana* var. *mangostana*, Clusiaceae) is often dubbed the “Queen of Fruits” (Fairchild, 1915). The fruit has gained great fame for the exquisite taste of its snow‐white flesh and is widely considered to be the finest fruit of the world (Almeyda & Martin, 1976; Dahlgren, 1947). A typical mangosteen fruit consists of usually six carpels, each with a segment of pulpy flesh, which collectively constitute the endocarp. Each segment encloses an ovule, but usually only one or two develop into seeds (asexually produced) in each fruit.

Mangosteen is the only *Garcinia* L. taxon widely cultivated at commercial scale for its fruit and has been introduced throughout the humid tropics. Southeast Asia is the major production area of mangosteen (Osman & Milan, 2006). Currently, mangosteen is also cultivated in the American and African tropics (Cruz, 2001; Lim, 2012; Murthy et al., 2018).

Despite a long history of cultivation, the domestication status of mangosteen, whether wild, domesticated, or semidomesticated (sensu C. Clement, 1999), is still being debated. Also, whether cultivated mangosteen has undergone substantial genetic adaptation to cultivated environments and consistent phenotypic changes under traditional cultivation management is still unknown. Under cultivation, has mangosteen evolved traits different from those of its wild ancestors, and if so, is it possible to retrace their evolutionary pathway? To answer these questions, we need to study the candidate wild progenitors, and to scrutinize the traits that may plausibly be expected to have been under selection during domestication.

We examine what little is known about these points. Some claims regarding the origin of mangosteen appear to be baseless (León, 1982; Zeven & de Wet, 1982), and theories of origin have been plagued with errors of fact and interpretation (Abdullah et al., 2012; Richards, 1990b). A thorough reappraisal is thus required. This review aims to provide a critical and in‐depth evaluation of previous notions about the origin of mangosteen, to bring new insights on the domestication of this species, and to identify knowledge gaps.

## 2 THE TAXONOMY AND DISTRIBUTION OF *GARCINIA* SECT. *GARCINIA*

The pantropical genus *Garcinia* comprises about 240 species (Stevens, 2001) of dioecious shrubs or trees (Sweeney, 2008) organized into 14 sections (Jones, 1980). The global distribution includes Central and South America, tropical Africa, Madagascar, the Mascarene islands, and throughout Southeast Asia to New Caledonia and northern Australia. Southeast Asia and Madagascar are the centers of species diversity (Sweeney, 2008). *Garcinia* is one of the most diverse tree genera in Asian tropical forests (Davies et al., 2005) but is poorly represented in the Americas (Stevens, 2007). The most recent global monograph for the genus is by Engler (1893, 1925). Many species have been added and the concepts of taxonomic sections remodeled over time.

**FIGURE 1:**
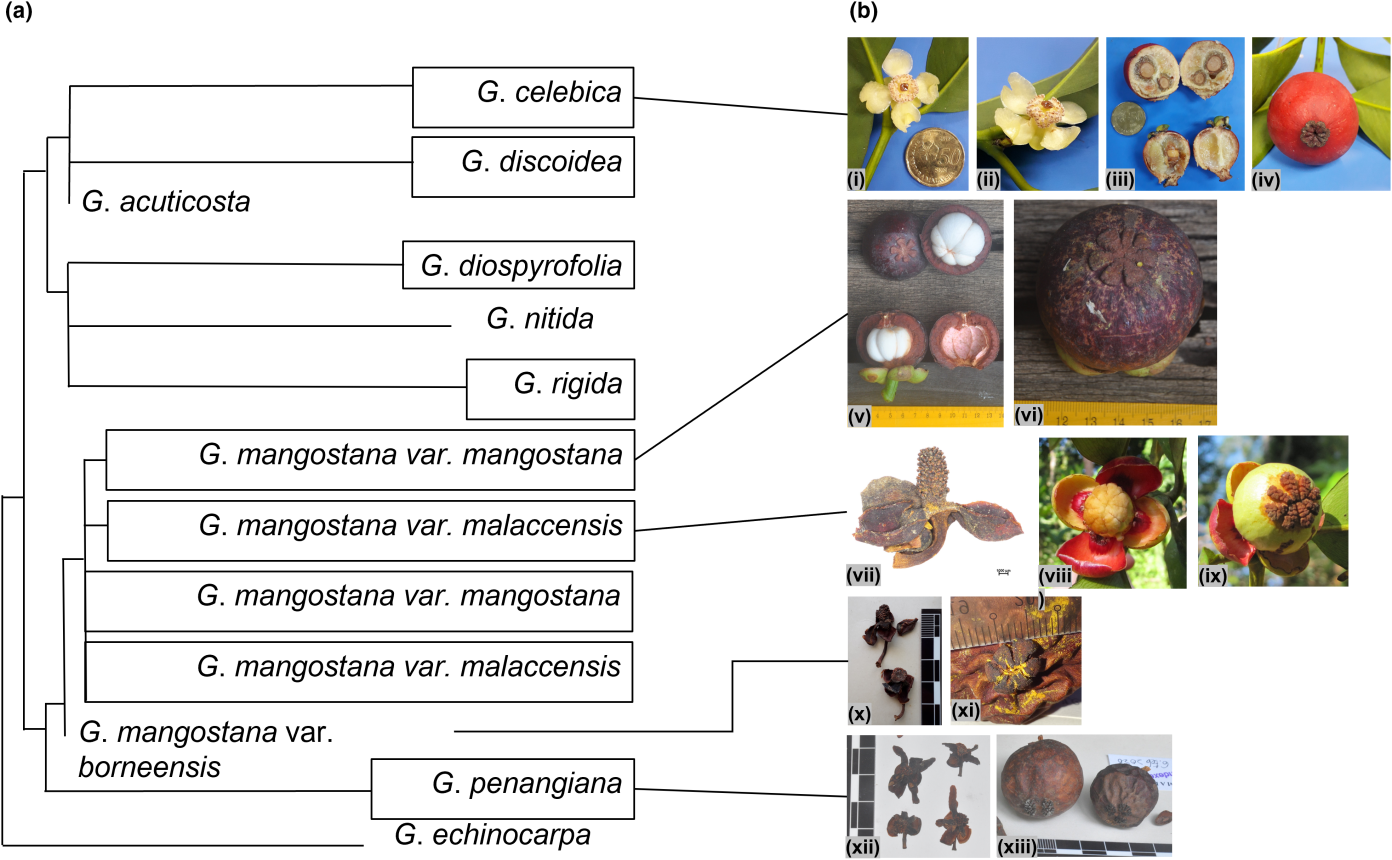
(a). Schematic phylogenetic tree adapted from Figure 5.3 in Nazre (2006) depicting the affinity between the taxa discussed in the text. Names in box denotes taxa with multiple accessions. (b). Photographs of live plants and herbarium specimens of selected taxa. *G*. *celebica*: (i) male flower (*YTL00014*, MPU) top view, (ii) side view, (iii) fruits (*YTL00016*, MPU) in transverse (TS) and longitudinal sections (LS), (iv) persistent stigma top view; *G*. *mangostana* var. *mangostana*: (v) fruits (*SAN161001*, SAN), exocarps and mesocarps dissected in TS and LS exposing the white pulpy edible endocarp, (vi) persistent stigma top view; *G*. *mangostana* var. *malaccensis*: (vii) male flower (*Collector unknown 6197*, SING) side view, (viii) female flower (*FRI72021*, KEP) top view, (ix) young fruit (*FRI72021*, KEP) persistent stigma top view; *G*. *mangostana* var. *borneensis*: (x) male flowers (*SAN61164*, KEP) side view, (xi) fruit (*AA1004*, WAN) persistent stigma top view; *G*. *penangiana*: (xii) male flowers (*La Frankie 2120*, KEP) side view, (xiii) fruits (*FRI27009*, KEP) persistent stigma tangential view. Photo credits: (viii) and (ix) by P. Wilkie (Royal Botanic Garden, Edinburgh), all others by the first author.

*Garcinia mangostana* belongs to *Garcinia* sect. *Garcinia*. Recently, Nazre et al. (2018) published a taxonomic revision of this section and recognized 13 species. Taxonomic concepts of this revision are followed here. Because we cite synonyms of accepted names (and the use of synonyms is duly noted) where necessary to facilitate the coherence of discussions, it is crucial for us to list the accepted names and their synonyms in Table 1. In order to provide an idea of the phylogenetic position of *G*. *mangostana* in sect. *Garcinia*, we present a schematic phylogenetic tree (Figure 1) adapted from Nazre's (2006) thesis. The closely related taxa repeatedly discussed in our study are illustrated with images.

**TABLE 1:** List of taxa of sect. *Garcinia* and their synonyms, adapted from Nazre et al., 2018.

	Current names	Synonyms	Geographical distribution
1	*Garcinia acuticosta* Nazre		Malay Peninsula
2	***G*** **.** ***celebica*** **L.**	*Brindonia celebica* (L.) Thouars; *G*. *basacensis* Pierre; ***G***. ***benthamii* Pierre**; ***G***. ***cornea* L**.**;** *G*. *fabrilis* Miq.; *G*. *ferrea* Pierre; ***G***. ***hombroniana* Pierre**; *G*. *jawoera* Pierre; *G*. *kingii* Pierre ex Vesque; *G*. *krawang* Pierre; *G*. *kurzii* Pierre; *G*. *porrecta* Anders.; *G*. *riedeliana* Pierre; *G*. *rumphii* Pierre; ***G***. ***speciosa* Wall**.; *Lignum corneum* Rumph.; *Mangostana celebica* Rumph.; *Oxycarpus celebica* (L.) Poir.; *Stalagmitis celebica* (L.) G. Don	Eastern India, Bangladesh, Indochina and throughout Malesia
3	*G*. *diospyrifolia* Pierre		
	var. *diospyrifolia*	***G***. ***opaca* King**; *G*. *opaca* var. *dumosa* Whitmore	Malay Peninsula and Borneo
	var. *cataractalis* (Whitmore) Nazre	*G*. *cataractalis* Whitmore	Malay Peninsula
	var. *minor* Ng ex Nazre		Malay Peninsula
4	*G*. *discoidea* Nazre		Malay Peninsula
5	*G*. *exigua* Nazre		Borneo
6	*G*. *harmandii* Pierre		Cambodia and Southern Vietnam
7	***G***. ***mangostana*** **L** **.**		
	**var** **.** ***mangostana***	*Mangostana garcinia* Gaertn.	Cultivated throughout the tropics
	**var** **.** ***malaccensis*** **(** **Hook** **.** ***f*** **.** **)** **Nazre**	***G***. ***malaccensis* Hook**. ***f***.; as *G. *malaccensis* var. *malaccensis* in Nazre (2006)	Sumatra, Malay Peninsula, Singapore, Borneo: Sarawak and Brunei
	**var** **.** ***borneensis*** **Nazre**	as *G. *malaccensis* var. *pseudomangostana* in Nazre (2006)	Borneo: East Coast of Sabah and Kalimantan
8	*G*. *nitida* Pierre		Borneo
9	*G*. *ochracea* Nazre		New Guinea
10	***G*** **.** ***penangiana*** **Pierre**		Sumatra, Malay Peninsula, Borneo
11	*G*. *rigida* Miq	*G*. *schefferi* Pierre	Sumatra, Indochina, Sulawesi (?), Maluku (?)
12	*G*. *sangudsangud* Nazre		Borneo
13	***G*** **.** ***venulosa*** **(** **Blanco** **)** **Choisy**	*Cambogia venulosa* Blanco; *G*. *cumingiana* Pierre	Philippines

Names mentioned in the article appear in bold typeface. Names marked with * are unpublished names that appeared in the thesis by Nazre (2006) and on determination slips on herbarium specimens. “Species with unknown status” in Nazre et al. (2018) originally listed by Jones (1980) as belonging to *Garcinia* sect. *Garcinia* are not included here.

**FIGURE 2:**
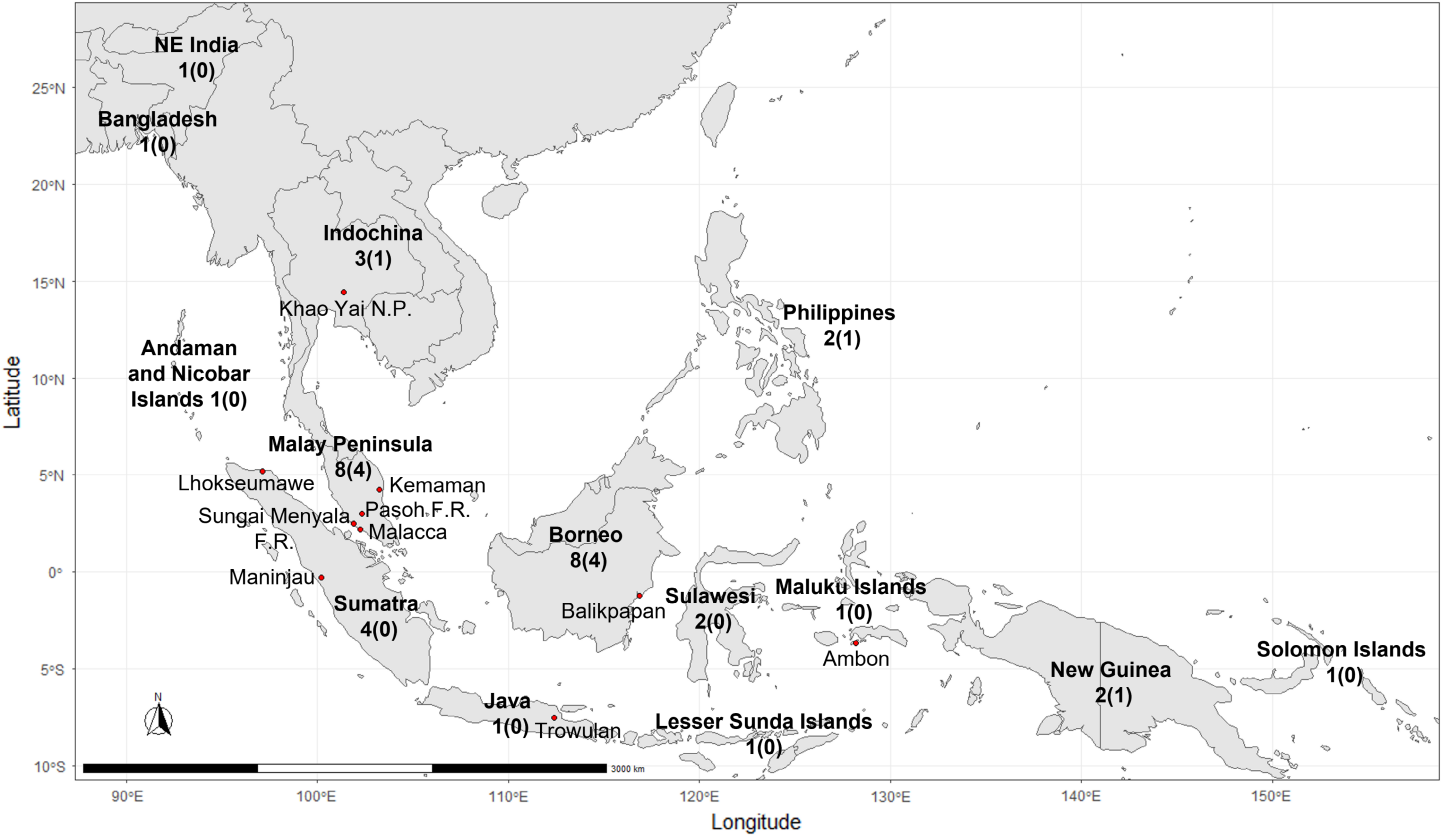
Geographical distribution of taxa of *Garcinia* sect. *Garcinia*. Bioregions appear in bold typeface. Sundaland includes Sumatra, Malay Peninsula, Java and Borneo. The numbers denote total numbers of species and varieties represented in each region; numbers in parentheses indicate the total number of taxa endemic to the region. *Garcinia mangostana* var. *mangostana* is not included in these tallies. Points in red denote localities mentioned in the text.

The geographical distribution of sect. *Garcinia* spans across eastern India, Bangladesh, Indochina, and throughout Malesia. We tabulated the geographical distributions of all species of the section in Table 1. Geographical distributions and species richness of different areas within the region are shown in Figure 2.

An important outcome of the revision by Nazre et al. (2018) that is especially pertinent to the understanding of the origin of cultivated mangosteen was their recognition of three varieties of *G*. *mangostana*. Cultivated mangosteen is recognized as *G*. *mangostana* var. *mangostana*, *G*. *malaccensis* was synonymized as *G*. *mangostana* var. *malaccensis*, and a new variety, *G*. *mangostana* L. var. *borneensis* Nazre, was described. Comparisons of these varieties are summarized in Table 2. Notably, texture of the persistent stigma surface is identified as a diagnostic character (Nazre et al., 2018) differentiating var. *mangostana* (smooth) from var. *malaccensis* (corrugated). However, Hambali and Natawijaya (2016) reported both character states in both var. *mangostana* and var. *malaccensis*.

**TABLE 2:** Comparison of the varieties of *Garcinia mangostana* (adapted from Nazre et al. (2018)).

Taxon	Presence of pistillodes in male flowers	Fruit shape	Fruit size	Fruit apex; persistent stigma shape, and surface texture	Cultivated or wild	Natural geographical distribution
*mangostana*	present*, *c*. 5 mm long.	ovoid or globose	4–7 cm across	rounded; circle, and smooth	cultivated; known as *manggis*; feral populations might exist	Malaysia and Indonesia
*malaccensis*	absent, or if present *c*. 2 mm	globose, ellipsoid or ovoid	3–4 cm across	acute to attenuate; circle, and rugose	both cultivated and wild; known as *mesta* in cultivation	Sumatra; Malay Peninsula; Borneo: Southwest Sarawak and Brunei
*borneensis*	absent, or if present *c*. 2 mm	globose	3–4 cm across	rounded; circle or oval, and smooth to slightly rugose	Wild	Borneo: East coasts of Sabah and Kalimantan

*Hambali and Natawijaya (2016) mentioned that the male flowers of var. *mangostana* described by Idris and Rukayah (1987) fell within the morphological plasticity of male flowers they observed in Sumatran populations of var. *malaccensis*.

It is crucial to point out that distributions of *G*. *mangostana* var. *malaccensis* and *G*. *penangiana* Pierre, which have often been confused (see below), overlap in Sumatra (Hambali & Natawijaya, 2016), the Malay Peninsula, and Borneo, and sympatric populations of the two exist. *Garcinia venulosa* (Blanco) Choisy was thought to be confined to Luzon Island, Philippines, but our examination of herbarium specimens showed that this species is also found on other islands of this country, namely Mindoro, Samar, and Mindanao. The wild relatives of mangosteen, namely *G*. *mangostana* var. *malaccensis* and var. *borneensis*, are confined to everwet rainforests in Sumatra, Malay Peninsula, and Borneo (Hambali & Natawijaya, 2016; Nazre et al., 2018). Field observations (T.L. Yao) on the wild populations of var. *borneensis* showed that trees were generally found along ephemeral streams in low‐lying flat land. Cultivated mangosteen also does best in environments with little water stress. Irrigation may be necessary during dry seasons (Osman & Milan, 2006).

The taxonomy of *Garcinia* is difficult (Sosef & Dauby, 2012), fraught with synonymies, and frequent misidentification of specimens. In this large genus, in which many species resemble one another in vegetative characters, identification to the species level is difficult when no reproductive organs are available. *Garcinia penangiana* is a species that has often been misidentified as *G*. *malaccensis*. The confusion thereby introduced has contributed to clouding the interpretation of the origin of mangosteen (section 3.2). Examinations of the specimens deposited in regional herbaria revealed that many specimens of *G*. *penangiana* were systematically misidentified as *G*. *malaccensis* by various collectors. Other species are also concerned by such confusion. “*Dr Kostermans has for some years now been labelling as* G. celebica *L*. *all specimens of this species‐group* [*G*. *penangiana* and other morphologically similar species] *in the herbaria he has visited*” (Kochummen & Whitmore, 1973). Erroneous identifications have had serious consequences for the interpretation of results used in constructing hypotheses about the origin of mangosteen (see section 3.2.1).

A few *Garcinia* species, namely *G*. *malaccensis*, *G*. *hombroniana* Pierre, and *G*. *venulosa*, have been regarded as wild species closely related to mangosteen based on morphological characters (Kochummen & Whitmore, 1973; Nazre et al., 2018; Ridley, 1922; Whitmore, 1973), and *G*. *malaccensis* was regarded as the most closely related species (Corner, 1997; King, 1890; Kochummen & Whitmore, 1973; Nazre et al., 2018; Whitmore, 1973). Assessment of morphological affinity between these taxa was mainly based on leaf characters. Herbarium specimens with flowers are generally hard to come by. Abdullah et al. (2012) hypothesized that *G*. *opaca* King is a possible hybrid parent of cultivated mangosteen based on their genetic analyses (section 3.2.1). *Garcinia opaca* has never been regarded as closely related to mangosteen based on morphological characters.

Prior to the taxonomic revision of Peninsular Malaysian Guttiferae (Clusiaceae) (Whitmore, 1973), *G*. *malaccensis* was deemed rare, being known only from Maingay's collections from Malacca (Figure 2) (*Kew Distribution No*. *149*, barcodes: K000380445, K000380446 and QR code: L.2416659) (Anderson, 1874; King, 1890; Ridley, 1922). Maingay's collections were first identified as *G*. *cornea* L. until J.D. Hooker in Anderson (1874) recognized them as constituting a new species and established *G*. *malaccense* (spelling variant). King (1890) mentioned that “*In its leaves*, *in the colour of its flowers*, *and in its 8‐lobed stigma*, *this resembles* G. mangostana.” On the contrary, Ridley (1922) mentioned that *G*. *malaccensis* “*closely resembles* Hombroniana, *but the stigma is lobed*.” Although the observations were only based on Maingay's collections with young flowers and flower buds—a solitary specimen of *G*.*malaccensis* consisting of a few duplicates—these early writings may have influenced subsequent authors' notions about the origin of mangosteen, especially the affinity between *G*. *hombroniana* on the one hand, and *G*. *mangostana* and *G*. *malaccensis* on the other. Whitmore (1973) indicated that *G*. *malaccensis* is locally common in several states in the Malay Peninsula and in Brunei. He benefited from examinations of the much‐expanded contemporary collections of *G*. *malaccensis* throughout the Malay Peninsula, especially from the east coast region, where many localities were explored for the first time in the late 1960s. These materials enabled him to expand and circumscribe the species concept of *G*. *malaccensis*.

Idris and Rukayah (1987) described the male flower of mangosteen as having numerous stamens “*surrounding a pistillode in a four‐angled mass*.” They also compared the morphology of male flowers of *G*. *mangostana* with that of *G*. *malaccensis* and *G*. *hombroniana*. However, Hambali and Natawijaya (2016) mentioned that the mangosteen male flowers described by Idris and Rukayah (1987) fell within the morphological plasticity of male flowers they observed in Sumatran populations of *G*. *malaccensis*. Nazre et al. (2018) drew attention to the morphological similarity of *G*. *venulosa* to *G*. *mangostana* var. *malaccensis* and to G. *mangostana* var. *borneensis*, in leaf and fruit traits, respectively. In our ongoing morphometric study of mangosteen and its wild relatives, we observed morphological intermediates between sympatric populations of *G*. *mangostana* var. *malaccensis* and *G*. *penangiana*.

The studies mentioned above defined the morphological characters of *G*. *mangostana* var. *mangostana* and the wild relatives. These findings enable us to identify the possible ancestral candidates of the cultivated mangosteen based on their morphological similarity.

## 3 DOMESTICATION OF *G*. *MANGOSTANA*

Cultivated mangosteen, like many trees in cultivation, was most probably domesticated incipiently, making the definition of cultivated, wild, or feral very challenging (Clement, 1999). The status of mangosteen, whether wild or cultivated, has long been debated. Views were contradictory in the early floristic accounts: “*native and cultivated*” (Anderson, 1874); “*wild and cultivated*” (King, 1890); and “*cultivated*… *not known in the wild state*” (Ridley, 1922) were descriptions of the states of cultivation. Corner (1952) opined that wild mangosteen populations were found in the then‐virgin forests in Kemaman (Figure 2) in Peninsular Malaysia, most likely vouchered as *Corner s*.*n*., 12 November 1935, Bukit Kajang Hillside, Kemaman, Terengganu, Peninsular Malaysia, barcode: SING0219178, which was later attributed to *G*. *malaccensis* (Whitmore, 1973) but to Corner's disagreement (Corner, 1997).

Ashton (1988) mentioned that *G*. *mangostana* occurs in the wild on the east coast of Peninsular Malaysia and in Borneo. According to F. Slik (pers. comm.), “wild” mangosteen morphologically similar to cultivated mangosteen and with similar‐tasting fruits was observed in a forest about 100 km inland from Balikpapan (Figure 2), Kalimantan, Indonesia. However, Lamb (2019) doubted that truly wild populations of *G*. *mangostana* exist in Borneo. Our field observations and herbarium specimens found that *G*. *mangostana* var. *borneensis* is locally common in the lowland forests on the east coast of Sabah, Malaysian Borneo.

Fruit trees, especially durian (*Durio zibethinus* L.) and mangosteen, are planted by the Temuan communities in the logged‐over timber production forest reserves in Peninsular Malaysia (T.L. Yao, pers. obs.). In the time before commercial logging, Temuan communities planted fruit trees in their old swiddens (Gianno & Bayr, 2009). An early record by Ridley (1893) reflected a similar observation: “*of fruits the usual Malay kinds are commonly cultivated, and the trees often persist long after the villages have been deserted and swallowed up in jungle. Durian…, mangosteen… are all plentiful.*” Herbarium specimens of mangosteen from Borneo, particularly in the river basin of Upper Rajang, Sarawak, may well have been obtained from remnant trees in the abandoned swidden plots. Thus, specimens collected in the forests might represent feral populations. Mangosteen present in agroforests in Maninjau (Figure 2), West Sumatra, is sourced from home gardens (Michon et al., 1986).

The descriptions above provide us a glimpse of occurrences of the wild relatives and the possible means of early domestication in cultivated mangosteen. In the following subsections, we present the historical record and linguistic evidence indicating the origin, and theories that have postulated the ancestors of cultivated mangosteen. Lastly, we discuss the possible domestication syndromes displayed in cultivated mangosteen.

### 3.1 What can the historical record and linguistic evidence tell us about the origin of cultivated mangosteen?

We combed through historical records to find indications about the origin of mangosteen. Written records chronicling the voyages of Cheng Ho (1371–1433), which took place between 1405 and 1433, indicated that fruits of mangosteen have been sold in marketplaces in the Malay Archipelago for at least 600 years (Gong, 2019 [originally published in 1434]; Ma, 2019 [originally published in 1416]). However, we do not know how different were these mangosteens from the mangosteen of today. Gong (2019) and Ma (2019) noted that mangosteen was among the fruits sold in the marketplaces in the royal capital of Majapahit (now Trowulan (Figure 2), East Java; Gomperts et al., 2012) and in Samudera‐Pasai (now Lhokseumawe (Figure 2), Aceh, Sumatra; Sulistiono, 2015). There is no information about whether the fruits were collected from forests or produced in orchards or home gardens. Notably, mangosteen was unequivocally transliterated as măng‐jí‐shì (莽吉柿) in Gong (2019) and Ma (2019) from *manggis*, a local name used consistently in coastal Borneo, Java, the west coast of the Malay Peninsula, and Sumatra throughout these few centuries. These regions correspond to the geographical extent of the core territories of the Majapahit Empire (1293–c. 1527 CE).

An annotated glossary “Hobson‐Jobson” (Yule & Burnell, 1886) detailed the origin of the name “mangosteen” and provided leads to a handful of early records about mangosteen documented in Western literature. de Orta et al. (1913 [originally published in 1563]) mentioned mangosteen and durian from Malacca in 1560's Portuguese Goa (de Orta et al., 1913) and implied that mangosteen was planted in Goa but had not yet yielded fruit at the time. The success of introduction, even if on a small scale and only for a short time, is shown by the mention of mangosteen in 1580's Portuguese India (van Linschoten et al., 1885). Followed by the burgeoning interest of early botanists in documenting exotic plant resources, especially those of economic value, accounts of mangosteen were presented in brief descriptions (Bauhin et al., 1650; Clusius, 1605; de Bondt, 1658) and listings (Bauhin, 1623). Mangosteen was deemed native to Malacca (Cardim, 1645), now a state in Peninsular Malaysia, and it was recorded as “*growing within the bush by the highway in Java*” in 1639 (de Mandelslo, 1669). Later, a fuller description of the plant appeared, based on the observation of mangosteen trees in Malacca (de Beze, 1692).

Until the 1690s, mangosteen was thus considered to have originated from the Malay Peninsula, Sumatra and Java, where the major ports of sea trade routes were located. At this juncture, mangosteen was also documented in Herbarium Amboinense (Rumphius, 2011 [originally published in 1741]), a catalogue of the plants of Ambon (Figure 2). In the text, mangosteen was considered foreign to Ambon but as being found in other Maluku islands (Figure 2), while Malacca, Sumatra, and western Java were stated to be the natural geographical range. Rumphius reported “*Only one or two trees that bear fruits reasonably well*” in Banda but stated that the trees “*do much better in Ternate and Gilolo*” (now Halmahera) owing to differences in soil properties. He made reference to Garcin's “de act. Paris. p. 431, Tab. 1”, which is most likely the original publication that was translated into the English version in Garcin (1733) annotated by Beekman in Rumphius (2011). Garcin (1733) published a detailed description of mangosteen based on trees grown in the Molucca (Maluku) Islands and stated that “*the tree originally grows in the Molucca Islands* … *it has been transplanted into the isle of Java and some few in Malacca*.” This work also reviewed earlier publications that mentioned mangosteen. Later, Linnaeus (1753) established the binomial name for mangosteen, *Garcinia mangostana*. Linnaeus (1754) honored Garcin by naming the genus after him. He was undoubtedly aware of Garcin's publication on mangosteen but gave Java as its geographical distribution (Linnaeus, 1753) instead of Maluku Islands. Remarkably, there is no evidence that Garcin had collected any specimen in the Maluku Islands (van Steenis‐Kruseman, 1950).

Concerning linguistic evidence, vernacular names of *G*. *mangostana* var. *mangostana* provide indications of its geographical origin. Cultivated mangosteen is locally known as *manggis*; another cultivar is called *mesta* (Table 2). The names *manggis* and *mesta* (or *masta*) do not necessarily denote distinct taxonomic entities, although their association with local dialects is traceable. The definition and usage of the names have possibly changed over time. *Manggis* is generally applied to var. *mangostana*. However, var. *mangostana* is known as *set'to* / *mes'tor* in the dialects of the east coast of Malay Peninsula, related to *mestar* of the Pattani‐Kelantan dialect (Yule & Burnell, 1886). *Mesta* is a recently popularized name used in reference to var. *malaccensis* in cultivation. This name is very likely a spelling variant of *set'to* / *mes'tor* / *mestar* that was attributed to var. *mangostana* on the east coast of Peninsular Malaysia and in southern Peninsular Thailand. Whitmore (1973) indicated “*manggis hutan*, (*m*. *burong*)” as the vernacular name of *G*. *malaccensis*. *Hutan* means forest, and *burong* (or *burung* in modern Malay Language) means bird, denoting the relatively small leaf and fruit. Examinations of the specimens found that *manggis hutan* is not exclusively applied to var. *malaccensis*, and *mangis burong* was actually applied to a specimen of *G*. *penangiana* (*Hashim 1185*, 9 May 1918, Penyabong, Johore, Peninsular Malaysia, barcode: KEP237257) misidentified as *G*. *malaccensis*.

Based on written historical records, inference from linguistic evidence and field observations, we speculate that mangosteen originated in the everwet zones of Southeast Asia, including Sumatra, Malay Peninsula, and Borneo, but excluding islands with a relatively pronounced dry season such as Java and Maluku.

### 3.2 What is the wild ancestor of *G*. *mangostana* var. *mangostana*?

In this section, we examine the taxa that have been proposed as the wild progenitors of mangosteen and critically examine the theories of its origin. Various hypotheses regarding the origin of mangosteen have been proposed (Table 3). Shaharudin et al. (2022) summarized and discussed these hypotheses but did not provide in‐depth interpretation. We especially discuss these hypotheses in light of findings from molecular biology accumulated over the past few decades.

**TABLE 3:** Summary of proposed hypotheses on the origin of cultivated mangosteen, listed chronologically.

Hypotheses	References
*Originated from *G*. *sylvestris* or *silvestris* (a spelling variant)	León, 1982; Zeven & de Wet, 1982
Hybrid of *G*. *hombroniana* and *G*. *malaccensis*	Richards, 1990b
Hybrid of *G*. *opaca* and *G*. *malaccensis*	Abdullah et al., 2012
Hybrid of different varieties of *G*. *malaccensis*	Nazre, 2014
Superior selections from female trees of *G*. *malaccensis*	Nazre, 2014
Selective cultivation of wild relatives through forest‐*dusun* interface	This article

*G. *sylvestris* or *silvestris* (a spelling variant) represents an unpublished species name; a thorough search in the Bogor Herbarium, Java, Indonesia failed to find any corresponding specimen.

#### 3.2.1 Mangosteen is a hybrid of two wild relatives

The hypothesis that *G*. *mangostana* arose from an allotetraploid hybrid between *G*. *hombroniana* and *G*. *malaccensis* (Richards, 1990b) has been cited in numerous horticultural monographs (Orwa et al., 2009; Osman & Milan, 2006; Verheij, 1991). Based on cytogenetic findings and morphological comparisons, Richards (1990b) posited that *G*. *mangostana* (2 *n* = 90?) arose from hybridization between *G*. *hombroniana* (2 *n* = 48) and *G*. *malaccensis* (2 *n* = 42–43?). Richards (1990b) conducted chromosome counts of *G*. *hombroniana*, whereas the count for *G*. *malaccensis* was cited from an unpublished Ph.D. thesis (Ha, 1978). Several subsequent molecular‐genetic studies have examined this hypothesis (Nazre, 2014; Sinaga et al., 2007; Sobir et al., 2009; Sulassih & Santosa, 2013; Yapwattanaphun et al., 2004). Richards (1990b) did not carefully consider the occurrences of aneuploidy in cultivated mangosteen that predated his study (Table 4).

**TABLE 4:** Chromosome counts of taxa in sect. *Garcinia* species.

Taxon	2 n	Reference	Sampling region	Cultivated or wild
*G*. *benthamii*	48	Tixier, 1960	Laos	Wild
*G*. *benthamii*	96	Chennaveeraiah & Razdan, 1980	India (?)	Wild
*G*. *hombroniana*	48	Richards, 1990a	Peninsular Malaysia	Wild, planted in botanic garden
*G*. *malaccensis*	48, 72	Hambali & Natawijaya, 2016	Sumatra	Wild
*G*. *mangostana*	*c*. 76	Krishnaswamy & Raman, 1949	Kerala, India	Cultivated
	96	Chennaveeraiah & Razdan, 1975; Tixier, 1960	India (?); Laos	Cultivated
	*c*. 88–90	Ha, 1978	Peninsular Malaysia	Cultivated
	*c*. 76, 96	Zeven and Wet 1982 (synthesis of findings)		Cultivated
	56–76	Soepadmo, 1989 (synthesis of findings)		Cultivated
	120–130	Othman & Tindall, 1995 (synthesis of findings)		Cultivated
	96	Hambali & Natawijaya, 2016	Java	Cultivated
	74–110	Midin et al., 2018	Peninsular Malaysia	Cultivated
*G*. *opaca*	*c*. 68	Ha, 1978	Peninsular Malaysia	Wild
*G*. *penangiana* (as *G*. *malaccensis*)	*c*. 42–43	Ha, 1978	Peninsular Malaysia	Wild
*G*. *speciosa*	*c*. 55	Krishnaswamy & Raman, 1949	Myanmar and Andaman Islands	Wild

The proposed close affinity between *G*. *malaccensis* and *G*. *mangostana* (Corner, 1997; King, 1890; Kochummen & Whitmore, 1973; Nazre, 2006; Nazre et al., 2018; Whitmore, 1973) has been repeatedly supported in molecular‐biological studies using various markers, that is, ITS (Nazre, 2014; Yapwattanaphun et al., 2004), AFLP (Sobir et al., 2009), RAPD (Sinaga et al., 2007), and ISSR (Sulassih & Santosa, 2013). However, Abdullah et al. (2012), Nazre (2014), Sandø et al. (2005), and Sobir et al. (2009) questioned the conclusion of a close genetic relationship between *G*. *hombroniana* and *G*. *mangostana*. All these studies demonstrated that *G*. *hombroniana* is only distantly related to mangosteen, in disagreement with Whitmore's (1973) opinion, based on morphological affinity, especially in vegetative characters, that *G*. *hombroniana* is one of the “close allies” of mangosteen.

Another twist cropped up when Nazre (2014) pointed out that the cytological information for *G*. *malaccensis* alluded to in Richards (1990b) was actually based on a misidentification of *G*. *penangiana* sampled from Pasoh Forest Reserve (F.R.) (Figure 2), Peninsular Malaysia. Richards (1990b) cited Ha (1978), repeating that author's erroneous identification. As mentioned in Section 2, many specimens of *G*. *penangiana* have been misidentified as *G*. *malaccensis*, causing confusion. Unfortunately, we could not locate the voucher specimen no. 85 cited by Ha (1978) among the herbarium specimens we examined, but confirmed that other specimens collected by Ha in the same site in the same period were all *G*. *penangiana*. Dysploidy, or varying chromosome number counts, in mangosteen (Table 4) hampered emergence of a convincing interpretation of hybridization history based on chromosome counts.

Abdullah et al. (2012) conducted a study using various genetic markers, namely nuclear ribosomal internal transcribed spacer (ITS), *trn*L, *acc*D‐*psa*L, and six microsatellite markers to elucidate the genetic variation within mangosteen and to infer the ancestry of mangosteen. The six microsatellite markers amplified in all five accessions of *G*. *mangostana* (four from Peninsular Malaysia and one from Sarawak) were monomorphic. On the contrary, polymorphism in microsatellite markers was observed among *G*. *malaccensis* (two accessions) and among *G*. *hombroniana* (three accessions). However, of the two accessions of *G*. *malaccensis*, that from Pasoh F.R., Peninsular Malaysia is most likely a *G*. *penangiana* misidentified as *G*. *malaccensis*, although the accession from Sungai Menyala F.R. (Figure 2) is correctly identified. *Garcinia mangostana* accessions shared allele sizes in microsatellite markers with *G*. *malaccensis* and *G*. *opaca*, but not with *G*. *hombroniana*. Topology of the most parsimonious trees from analyses of ITS, *trn*L, and *acc*D‐*psa*L sequences showed *G*. *malaccensis* to be a possible progenitor of mangosteen. Abdullah et al. (2012) included one out of the two accessions of *G*. *malaccensis* in their phylogenetic analyses, but did not disclose the locality, whether from Pasoh F.R. or Sungai Menyala F.R., of the accession analyzed. It is thus possible that their conclusions on phylogenetics finding are also based on a misidentified sample. The placement of *G*. *opaca* next to *G*. *mangostana* and *G*. *malaccensis* (Abdullah et al., 2012) led the authors to propose *G*. *opaca* as a likely parent. However, other than *G*. *mangostana*, only four of the 12 other species within sect. *Garcinia* (Nazre et al., 2018), namely *G*. *malaccensis*, *G*. *penangiana*, *G*. *opaca*, and *G*. *hombroniana*, were included in the analyses. *Garcinia hombroniana* was unequivocally rejected as a possible progenitor based on microsatellite markers and molecular‐phylogenetic results. Thus, *G*. *malaccensis* and *G*. *opaca* appeared as cultivated mangosteen's closest relatives.

The results obtained by Abdullah et al. (2012) and the comparison of phylogenetic trees based on one nuclear marker (ITS), or on cpDNA markers, do not allow any conclusion about the occurrence of hybridization among species of *Garcinia* sect. *Garcinia*. Incongruences between nrDNA and cpDNA phylogenetic trees can be related to hybridization events involving cytoplasmic captures but can also be explained by patterns of incomplete lineage sorting. In addition to sample misidentification and misinterpretation of the findings, this study suffered from very small sample size.

#### 3.2.2 Mangosteen derived solely from *G*. *malaccensis*

Yapwattanaphun et al. (2004) were first to demonstrate the phylogenetic relationships between mangosteen and 16 wild species using ITS sequences. Both unordered parsimony and neighbor‐joining analyses pointed to *G*. *malaccensis* as the closest relative of mangosteen. In more recent studies (Hambali & Natawijaya, 2016; Nazre, 2014), *G*. *malaccensis* is proposed as the sole progenitor of mangosteen.

Nazre (2014) attempted to infer the progenitor of mangosteen based on ITS sequences with a focused sampling including only species that had been hypothesized by previous studies to be putative progenitors of mangosteen. Analyses revealed that mangosteen and *G*. *malaccensis* shared >99% of their sequences. Mangosteen and *G*. *malaccensis* accessions formed a monophyletic clade (bootstrap value = 81%) and *G*. *penangiana* accessions appeared as the sister clade. However, the use of a single and short marker gives only limited insight into the extent of divergence among species.

Hambali and Natawijaya (2016) posited that mangosteen arose from *G*. *malaccensis* through autopolyploidization. This hypothesis was based on cytological study of a planted population of *G*. *malaccensis* (2 *n* = 2x = 48), sourced from Sumatran forests, and cultivated mangosteen (2 *n* = 4x = 96). The chromosome counts of the latter concurred with the findings of Chennaveeraiah and Razdan (1975) and Tixier (1960). The study also revealed the occurrence of triploid forms (2 *n* = 3x = 72) in the wildlings grown in the garden, and the authors were able to produce the triploid form by artificial pollination of *G*. *mangostana* with *G*. *malaccensis* pollen in cross‐breeding trials. The results of their crossing experiments demonstrated that mangosteen is capable of reproducing sexually and showed that there is no breeding barrier between wild *G*. *malaccensis* and cultivated mangosteen. These results suggest that gene flow might take place between wild relatives and cultivated populations. Based on their results, Hambali and Natawijaya (2016) proposed a pathway for the origin of mangosteen. First, on individuals of female *G*. *malaccensis* that were able to reproduce apomictically, diploid meristematic vegetative tissues underwent spontaneous ploidy duplication and eventually developed into a tetraploid branch sport. Later, the tetraploid branch sport produced fruits with tetraploid asexual seeds and gave rise to *G*. *mangostana*. They posited that variation, which developed later in the apomictic tetraploid mangosteen populations, arose through (somatic) mutation and is of secondary importance.

Again, extensive variation of chromosome numbers in mangosteen (Table 4) precludes drawing convincing conclusions about the hybrid origin of mangosteen based on chromosome counts. The findings of Hambali and Natawijaya (2016) do not provide empirical evidence for an autotetraploid origin of mangosteen. Direct cytogenetic observation of male meiosis of a triploid produced from the crossing, if available, should be made. Also, the pattern of chromosome pairing could help ascertain whether there are two or four genomes of *G*. *malaccensis* in *G*. *mangostana* (pers. comm., A.J. Richards). Recently, an unpublished Ph.D. thesis (Midin, 2018) reported results of Genomic in situ Hybridization (GISH), using *G*. *malaccensis*, *G*. *penangiana*, *G*. *hombroniana*, and *G*. *opaca* as probe and blocking DNA in GISH analyses. Based on the results, the author postulated that mangosteen and *mesta* originated from *G*. *malaccensis*.

Knowing that the closest wild relatives of mangosteen are confined to Sundaland (Figure 2) and *G*. *mangostana* var. *malaccensis* (which, again, does not occur in Maluku) is its likely progenitor, the presumption that mangosteen originated from Maluku Islands, as suggested by Garcin (1733), also cited by Baleshwor Sharma et al. (2013), is doubtful. However, we do not attempt to define a specific center of origin here. Multiple selections of wild progenitor(s) from widespread populations throughout the natural range of wild relatives might have occurred in Sundaland, a circumstance Harlan (1992) termed “diffuse origin”. Mangosteen might have originated and diffused from its geographic origin(s) well before any written record existed.

### 3.3 The possible domestication syndromes of mangosteen

Assessment of the various hypotheses on the origin of mangosteen in light of morphological comparisons and new genetic information supports the conclusion that *G*. *mangostana* var. *malaccensis* is the sole progenitor of mangosteen. Below, we compare the traits of mangosteen and its wild relatives, focusing particularly on *G*. *mangostana* var. *malaccensis*. We examine the proximate mechanisms that might have led to changes in genetic and breeding system traits in mangosteen and the selective pressures that may have favored trait evolution.

#### 3.3.1 Do male trees occur in mangosteen?

Dioecy accounts for 5%–6% of angiosperms (Renner, 2014) and is prevalent in *Garcinia* species (Ha et al., 1988; Thomas, 1997). Whether male trees exist in mangosteen has been a subject of debate since the 1830s (Horn, 1940; King, 1890; Ridley, 1922; Roxburgh, 1832). Pierre (1882) and Chevalier (1919) reported conflicting observations on the occurrence of male trees within mangosteen plantations in Cochin–China (now southern Vietnam), where mangosteen was introduced as a fruit crop. Pierre examined about 1500 trees yet could not find a male plant. Chevalier (1919) stated that “*all Garcinia trees are dioecious but the female plants of mangosteen also bear male organs so that all the male plants in a garden can be removed without inconvenience*. *This is what the natives do*, *and in the first years of flowering*, *they cut down the plants that do not produce fruits or hardly any*.” Chevalier did not state whether the male organs on female plants were present in (i) separate male and female flowers on the same plant (that is monoecy), or (ii) in hermaphrodite flowers. He apparently had no knowledge of Sprecher's (1919a, 1919b) publications on apomictic reproduction of mangosteen.

Descriptions of the practice of culling male trees by Chevalier (1919), which were later cited in Burkill (1966), are therefore dubious. To the best of our knowledge, no report of a practice of culling of male trees has appeared in other literature, nor is this practice known from any commercial orchard. However, the culling reported by Chevalier (1919) might have been a practice to eradicate those female trees that took a longer time to become productive.

Male organs on mangosteen flowers observed by Chevalier (1919) were most likely staminodes, the reduced male organs. Staminodes are most easily observed in flowers that have shed their corolla or on young fruits, where they are attached around the base of the ovary. Staminodes are present in the functionally female flowers of mangosteen, where they produce no viable pollen (Lim, 1984). We also observed staminodes in other taxa in sect. *Garcinia*, viz. *G*. *mangostana* var. *malaccensis*, *G*. *penangiana*, and *G*. *venulosa*. Hence, the presence of staminodes on functionally female flowers of mangosteen is unlikely to be a trait of mangosteen that evolved under domestication. Idris and Rukayah (1987) reported a mangosteen tree bearing male flowers in Peninsular Malaysia. The authors mentioned that the flowers were functionally male with a stamens mass surrounding a pistillode (Idris & Rukayah, 1987, Plate 2–4). They describe and provide scanning electron microscope images of the pollen grains, but gave no information on their viability.

Very few data exist on sex ratio in populations of wild *Garcinia* spp. During a flowering event, a field census of *G*. *penangiana* (misidentified as *G*. *malaccensis*) in Pasoh F.R. conducted by Thomas (1997) enumerated 31 staminate and 48 pistillate individuals. Male trees have been reported to be extremely rare in *G*. *parvifolia* (Miq.) Miq. No male trees of *G*. *parvifolia* were found in Pasoh F.R. (Figure 2), Peninsular Malaysia (Ha et al., 1988); nevertheless, female trees produced fruits. Thomas (1997) speculated that the low proportions of male trees observed in some *Garcinia* spp. may have driven natural selection favoring apomictic individuals. Having said that, we know neither what causes low proportions of male trees in some *Garcinia* spp., nor the sex ratio in populations of *G*. *mangostana* var. *malaccensis* and var. *borneensis*.

#### 3.3.2 Apomixis

Agamospermy may be frequent in *Garcinia* (Sweeney, 2008), and somatic embryogenesis has been demonstrated in cultivated mangosteen (Lim, 1984; Sprecher, 1919a, 1919b). Asexual seeds are formed by an adventive embryo originating from a somatic cell in the epithelium of the inner integument layer of the ovary (Sprecher, 1919a, 1919b). According to Horn (1940) and Lim (1984), mangosteen does not produce viable pollen. However, Horn's observations were based on shriveled flowers from two trees in an experimental station and may not constitute robust evidence. Lim (1984) conducted a bagging experiment to test whether pollen is required for seed production in mangosteen. On 50 bagged flowers, staminodes were left intact. On another 50 flowers, staminodes were removed prior to bagging. She found that flowers in both treatments set fruit, showing that fruit set did not require the presence of pollen. Fruit set in flowers from which staminodes had been removed (20%) was lower than in intact flowers (53%), owing probably to damage inevitably caused by manipulation during removal of staminodes. Unfortunately, there is no empirical genetic evidence for apomictic reproduction, e.g., highly similar or even identical multi‐locus microsatellite genotypes between a mother tree and its progeny.

Hambali and Natawijaya (2016) reported that they successfully pollinated *G*. *mangostana* var. *mangostana* flowers with *G*. *mangostana* var. *malaccensis* pollens in garden experiments and obtained viable seeds. The resulting seeds developed into triploid seedlings, suggesting that they were sexually produced, in contrast to the tetraploid seedlings that result from apomictic reproduction of mangosteen (Hambali & Natawijaya, 2016). The results of these experiments suggest that mangosteen is facultatively apomictic, capable of sexual reproduction when a suitable source of viable pollen is available. From these findings, we know that at least some *G*. *mangostana* var. *malaccensis* produce viable pollen. If this is a general trait of this wild species, it might be inferred that male sterility evolved in cultivated mangosteen. However, given the paucity of studies of the reproductive biology of *G*. *mangostana* var. *malaccensis* and other wild species, we cannot exclude the possibility that pollen viability is a variable trait in the wild ancestor and that male sterility may thus have preceded the evolution of cultivated mangosteen. Systematic comparative studies of pollen viability in wild relatives are crucial to validate our inference.

These findings suggest parallels with the evolution of parthenocarpy in banana (*Musa* L. spp.). Domesticated bananas produce no functional stamens, and produce seedless fruits parthenocarpically. However, some “primitive” cultivars are facultatively parthenocarpic: they can produce normal seeds through sexual reproduction if they receive viable pollen from wild relatives. Later in evolution under domestication, “advanced” banana cultivars became obligately parthenocarpic (McKey et al., 2010). Replication of the cross‐pollination experiments and genotyping of parents and progeny would resolve key questions about the biology and breeding of mangosteen, such as its ability to produce sexual seeds if supplied with an appropriate pollen source.

Information on breeding biology of wild *Garcinia* spp. is scarce, but some are known to form seeds parthenogenetically. Seeds are formed by an adventive embryo originating from a somatic cell in the epithelium of the inner integument layer of the ovary in *G*. *kydia* Roxb. and *G*. *treubii* Pierre (Treub, 1911). “Embryonic buds” formed on the inner integument were also observed in *G*. *parvifolia* and *G*. *scortechinii* King but do not develop into adventive embryos. In *G*. *parvifolia*, the unfertilized egg cell develops into an embryo (Ha et al., 1988). Mesta, the cultivated *G*. *mangostana* var. *malaccensis*, is likely capable of producing fruits apomictically, as our field observations and interviews with growers found no male individuals in cultivation. The capability of both cultivated and wild *G*. *mangostana* var. *malaccensis* and var. *borneensis* to reproduce apomictically could be ascertained with empirical studies, for example, bagging experiments on wild trees with staminodes removed, coupled with studies of embryonic development. These studies would confirm whether plants of these taxa are capable of apomictic reproduction.

#### 3.3.3 Polyploidy

The postulated earliest event of evolution of polyploidy in *Garcinia* traces back to 86.3 MYA (Landis et al., 2018) and the dating of the whole‐genome duplication event was inferred from analyses of the 1000 Plants (1KP) transcriptome data. Carman (1997) suggested that the base number of chromosomes is eight for *Garcinia*. The chromosomes of *Garcinia* are small and difficult to count (Midin et al., 2018; Richards, 1990b). A number of *Garcinia* species variably display diploidy, dysploidy and polyploidy (Table 4). The African species *G*. *kola* Heckel is most probably hexaploid, given results obtained on its genome size, chromosome numbers and SSR profiles (J. Duminil, unpublished data). Chromosome numbers reported for *G*. *benthamii* Pierre are 48 (Tixier, 1960) and 96 (Chennaveeraiah & Razdan, 1975), indicating possible diploidy and tetraploidy in the species. Chromosome number of *G*. *mangostana* var. *malaccensis* was first published only recently (Hambali & Natawijaya, 2016); diploidy and natural triploidy were observed. Polyploidy thus exists within species of sect. *Garcinia* and is not exclusive to mangosteen. The variation in chromosome number is notably greater in mangosteen than in related taxa, but this might be due to inadequate study of the wild taxa.

Polyploidy is well‐documented in mangosteen (Hambali & Natawijaya, 2016; Midin et al., 2018). Matra et al. (2016) demonstrated that mangosteen accessions displayed more than two alleles per locus in microsatellite markers in population‐genetic studies. Midin et al. (2018) reported highly variable chromosome numbers, 74–110, in an analysis of 20 metaphase spreads, demonstrating dysploidy/aneuploidy in mangosteen.

Nazre (2014) implied autopolyploidy, rather than allopolyploidy, in mangosteen with findings of high genetic similarity between var. *mangostana* and var. *malaccensis*. Also, Nazre (2014) detected identical substitution sites and indels in ITS sequences in one sample each of *mesta* (cultivated var. *malaccensis*) and wild var. *malaccensis*. Midin et al. (2018) performed rDNA‐Fluorescence in situ Hybridization (FISH) analyses on mangosteen and mesta chromosomes and reported four hybridization signals, indicating tetraploidy in both cultivated entities. However, it must be pointed out that several loci of rDNA repetitive clusters may occur even in some diploid species, and that polyploid species with a single rDNA locus can also be found (Garcia et al., 2017). Thus, the number of FISH signals does not allow inference of ploidy level.

Polyploidy in mangosteen is readily maintained by apomictic reproduction, which avoids potential difficulties in chromosome pairing during meiosis. However, whether apomixis is developmentally linked to autopolyploidy, as suggested for other plants (Grimanelli et al., 2001), or is instead an independently evolved phenomenon, remains unknown. We do not have enough information about the development of polyploidy in mangosteen or its wild relatives to assign them to any of the models presented by Hojsgaard and Hörandl (2019).

Differences in ploidy level within a species might be reflected in phenotypic variations (He et al., 2018; Ramsey & Ramsey, 2014). Studies found that *G*. *mangostana* var. *mangostana* is a tetraploid (Matra et al., 2016); diploids and triploids occur in var. *malaccensis* (Hambali & Natawijaya, 2016). Midin et al. (2018) reported tetraploidy in mesta, the cultivated var. *malaccensis*. Ploidy level of var. *borneensis* is unknown. Due to ploidy variation and the presence of aneuploids (Midin et al., 2018), demarcating these entities using morphological characters remains a conundrum.

#### 3.3.4 Genetic variation

Progeny produced by apomixis are in theory genetically identical to the maternal plant (Hand & Koltunow, 2014). Apomixis in mangosteen led to the supposition that only limited genetic variation would be found within mangosteen populations (Horn, 1940), provided that all domesticates derived from a single original clone. However, genetic variation was detected by various markers in mangosteens grown in Australia (Ramage et al., 2004; Sandø et al., 2005) and Indonesia (Mansyah et al., 2003; Matra et al., 2016). Matra et al. (2016) characterized the genetic diversity of five cultivated mangosteen populations in Java using microsatellite markers, and found an average genetic diversity (*H*_T_) of 0.444.

It is essential to identify the sources of the observed variation. Genetic variation in mangosteen might arise from three sources: (i) multiple selections of wild progenitors originating from independent events of sexual reproduction; (ii) heritable somatic mutation; and (iii) facultative apomixis that allows gene flow from wild populations. Sandø et al. (2005) and Nazre (2014) posited that variation in cultivated mangosteens arose from several initial events of adoption of sexually produced progenitors with considerable genetic differences among them. These could be ancient adoptions. Extensive population‐genetic studies that include a considerable number of accessions of mangosteen and of its putative wild relatives might allow a test of this hypothesis.

Are somaclonal mutations the major source of variation in mangosteen, as Richards (1996) concluded for *Taraxacum* L.? Apomixis and other forms of asexual propagation are expected to lead to increased heterozygosity, because as somatic mutations are incorporated and transmitted, it is very unlikely that the same mutation will occur at the same locus on both chromosomes (Balloux et al., 2003). Variation at neutral loci may be notable in mangosteen, because this type of variation increases during somaclonal evolution. Microsatellite markers often evolve by stepwise mutations, so that if allele size diversity is small in relation to allele number diversity, this indicates recent differentiation (Hardy et al., 2003; Léotard et al., 2009), consistent with diversification via somaclonal mutation. Findings by Samsir et al. (2016) demonstrated that Simple Sequence Repeats (SSRs) alleles of mangosteen show little size variation, suggesting that they diversified via somaclonal mutations. However, considering the level of genetic variation observed by Matra et al. (2016) in Javanese *G*. *mangostana* var. *mangostana*, it is unlikely that somaclonal mutations were the sole source of variation.

Hambali and Natawijaya (2016) demonstrated that cross‐pollination between mangosteen and wild *G*. *mangostana* var. *malaccensis* is possible. In this context, gene flow would be unidirectional from wild var. *malaccensis* to cultivated mangosteen, with variation contributed only by pollen donors. Sexuality generally exists in perennial apomictic plants (Richards, 2003), and events of sexual reproduction in a generally apomictic line, although infrequent, can have a disproportionately large effect on genetic variability (Halkett et al., 2005).

Based on both empirical findings and theoretical arguments, the expectation that genetic variation is absent in mangosteen owing to its apomictic reproduction should be abandoned. However, the pattern of genetic variation in apomictic species is different from that in sexually propagated species.

## 4 HYPOTHESIS: DOMESTICATION AT THE FOREST‐*DUSUN* INTERFACE

Mangosteen is attractive to humans owing to its tasty fleshy fruits. As for other fleshy‐fruited trees, its cultivation and eventual domestication may be seen as a process of humans taking over the function of seed dispersal from other frugivorous mammals. Studying *G*. *benthamii* in Khao Yai National Park (N.P.) (Figure 2), central Thailand, McConkey et al. (2015) documented the effectiveness of various mammals as seed dispersers of wild large‐fruited *Garcinia* spp. Like *G*. *mangostana*, *G*. *benthamii*, which has been synonymized with *G*. *celebica* (Nazre, 2010), belongs to sect. *Garcinia*. The fruits are available on the trees for about four weeks and remain fresh for at least four weeks after falling to the ground. McConkey et al. (2015) identified gibbons, which swallowed and defecated seeds, as the main arboreal consumers. Macaques fed almost equally from the tree canopy and from the ground and spat out the seeds. Squirrels, sambar deer, and barking deer consumed the fruits but destroyed the seeds. Elephants were also recognized as dispersers of *G*. *benthamii*, as was evident from intact seeds and partly digested fruits found within dung piles. Of all species of sect. *Garcinia*, *G*. *mangostana* possesses the thickest mesocarp (the inedible tissue enclosing the edible white flesh and the seeds inside). However, nimble‐fingered primates such as gibbons and macaques would not be deterred from consuming the flesh and in turn, dispersing the seeds.

Humans doubtless have long consumed the fruits of edible *Garcinia* spp. when chanced upon. “*A few of the species with better developed fruits have been brought into cultivation by primitive man*. *The mangosteen*, *the Asam Gelugor* (G. atroviridis *Griff*. *ex T*.*Anderson*), *the Kechupu* (G. prainiana *King*), *the Mundu* (G. dulcis (*Roxb*.) *Kurz*) *and the village Kandis* (G. cowa *Roxb*.) *are common village fruit‐trees*, *… they are all native in the forest”* (Corner, 1997). Early cultivators could have modified the geographic ranges of a few edible *Garcinia* spp., without substantially changing their genetic constitution. However, farmers' practices, and adaptations to the new environmental conditions created by farmers, could lead to genetic change. Farmers typically grow mangosteen in subsistence orchards, which are often located close to forest. These orchards are termed *dusun*, a Malay word that also means “a secluded hamlet” (Dewan Bahasa dan Pustaka, 2021). Mangosteen might have first arisen in the “forest‐*dusun* interface.”

What processes affected mangosteen populations in this interface? Our current knowledge about the biology of mangosteen and its wild relatives does not allow us to propose detailed hypotheses about the evolutionary ecology of mangosteen domestication. Knowledge about the breeding system of the wild relatives is especially inadequate. Based on our review of past findings, we speculate on domestication processes to frame testable hypotheses.

Initially, the early cultivators most likely grew plants from seeds of multiple individuals of mangosteen's wild progenitors, either intentionally planted or dropped and left to germinate along trails, in home gardens etc. These early introductions from the forest most likely occurred independently throughout the natural distribution of mangosteen's progenitors in Sumatra, the Malay Peninsula and Borneo, and over spans of time that may have varied among these regions. With the beginning of cultivation, the progenitors of mangosteen entered a phase that we call the “forest‐*dusun* interface.” The trees were grown in the mixed shifting/sedentary *dusun* located in proximity to or even within mature forests.

Two scenarios can be envisaged for domestication in this cultural–ecological setting. In the first scenario, apomixis, and perhaps polyploidy, emerged during domestication. At the forest‐*dusun* interface, occasional gene flow between the planted mangosteen trees and wild individuals was possible. Gene flow during this phase might have happened in two ways. While genetic variation from the wild populations intermittently contributed to the genetic diversity of the planted trees when cross‐pollination occurred, gene flow from planted trees to the wild populations might also have occurred. Culling of male trees, which do not bear fruit, might have been practiced by cultivators in the forest‐*dusun* interface. Fruit production by planted trees would then have depended on pollen supplied by males from the wild compartment. As females became increasingly predominant in the planted compartment, and as mature forest and the wild compartment receded, mate limitation would have reduced fruit production by cultivated females. Under these conditions, females capable of apomictic production of fruits would have been favored by humans. Apomixis may have first been facultative, perhaps becoming obligate in some genotypes as decreasing pollen flow from wild males lowered the selective advantage to cultivated female trees of maintaining the ability to reproduce sexually. Polyploidy (tetraploidy) may have evolved independently of apomixis; or they might have been developmentally related consequences of a single set of underlying genetic changes, as seen in *Paspalum notatum* Flüggé, where simple chromosome duplication of diploid plants results in production of apomictic autotetraploids (Grimanelli et al., 2001). Alleles permitting apomixis are present in the diploids, and their expression is triggered by polyploidy. Whether apomixis in mangosteen is dependent on or independent of polyploidy, polyploidy may have conferred advantages under human selection (Zhang et al., 2019), which include increased vigor and reduced investment in sexual reproduction, enabling greater investment in producing organs of interest to humans such as fruit flesh (McKey et al., 2010).

In the second scenario, apomixis, predominance of female trees, and polyploidy (tetraploidy) already characterized the wild progenitors of mangosteen. As Hörandl and Hojsgaard (2012) pointed out, apomixis and polyploidy are often related in evolution. If the planting stocks were sourced exclusively from apomictic tetraploid wild populations, as proposed by Hambali & Natawijaya, 2016, only fruit‐bearing female trees existed, even in the wild compartment. Individuals in both the wild and the cultivated compartments may have varied in ploidy levels in the forest‐*dusun* interface. In this scenario, apomixis and polyploidy did not arise under domestication but were already traits of the plants selected for cultivation. Thomas (1997) suggested that apomixis could be advantageous for forest trees that typically occur at low population density.

How can we assess the plausibility of these two hypotheses? If *G*. *mangostana* var. *malaccensis* is truly the wild progenitor of cultivated mangosteen, the first scenario could be falsified, and the second scenario supported, if it were found that (i) female individuals predominate, even in wild populations; (ii) wild female trees are capable of apomictic reproduction; and (iii) wild individuals are tetraploid, not differing from mangosteen in this regard. There remains, however, the strong possibility that truly wild progenitors of mangosteen no longer exist and that “wild” populations of *G*. *mangostana* var. *malaccensis* are in fact feral, derived from individuals escaped from cultivation, or left in the subsistence farms subsequently swallowed by adjacent forests. In this case, *G*. *mangostana* var. *malaccensis* may bear a mixed genetic heritage, its genome bearing elements with gene constitutions of wild progenitors that no longer exist; while cultivated mangosteen and wild varieties other than var. *malaccensis* might represent a polyploid series with phenotypic variations. Testing this hypothesis would require cytological and genomic studies.

By either scenario, humans favored individuals with traits such as large, tasty fruits and the ability to produce them in the absence of male trees. Such individuals may have been positively selected by humans over time and widely propagated and diffused. In fact, fruits of cultivated mangosteen are larger than those of any other taxon in the section, including *G*. *mangostana* var. *malaccensis* (Nazre et al., 2018), but it is currently impossible to determine whether this difference is genetically based or represents phenotypic plasticity in response to more favorable conditions in cultivated environments. Mangosteen has been available in markets for at least 600 years, probably even earlier at seaports of the regional maritime kingdoms, where exchanges of forest products were common. Early planting stocks might have been acquired from different sources throughout Sumatra, the Malay Peninsula and Borneo at different times over a long period and gradually diffused to the entire Malay Archipelago.

The current practice of growing mangosteen in commercial orchards isolated from natural populations reduced or halted gene flow between mangosteen and wild relatives. In commercial orchards where mangosteen reproduces exclusively apomictically, genetic variations between mother tree and progeny, if detected, may be principally due to the integration of somatic mutations.

## 5 SYNTHESIS

We have clarified issues concerning misidentification of samples that have long plagued studies of the origin of cultivated mangosteen, in particular, analyses and observations based on *G*. *penangiana* misidentified as *G*. *mangostana* var. *malaccensis*. This case underlines the importance of taxonomic study as the cornerstone of biological research and domestication studies. In turn, taxonomic studies depend on systematic botanical surveys that assemble the materials necessary for evaluating character variation in closely related taxa and that collect fresh leaf tissues for DNA analyses that can complement morphology‐based taxonomy.

Most previous studies of patterns of genetic variation in mangosteen and its wild relatives based on microsatellites have suffered from small sample sizes and limited geographical representation. Inclusion of fewer than a handful of accessions of closely related wild relatives does not allow ascertaining the occurrence of gene flow between wild and cultivated populations, nor does it shed light on the origin of cultivated mangosteen. Studies of *Eucalyptus grandis* W. Hill ex Maiden indicate that to estimate expected heterozygosity with minimum variance, at least 64 individuals need to be genotyped for most microsatellites (Kirst et al., 2005). Furthermore, sampling of mangosteen and its wild relatives has been restricted to the particular geographical regions where the researchers were based. To enable credible and comprehensive conclusions regarding the origin of mangosteen, it is crucial to obtain numerous, geographically representative samples from all the countries spanning Sumatra, the Malay Peninsula, Java and Borneo. International collaboration is indispensable to this end.

*Garcinia mangostana* var. *malaccensis* and var. *borneensis* most probably form the core of mangosteen's wild‐growing primary gene pool. We could not definitively exclude the possibility that other species genetically related and morphologically similar to mangosteen, viz. *G*. *penangiana*, and *G*. *venulosa* (Nazre, 2006; Nazre et al., 2018), might also have contributed to the gene pool of mangosteen. Whether traceable gene flow occurs (or occurred in the past) between mangosteen and its wild relatives in nature, and if so, how pervasive it is or was, are unknown. Another pertinent question is whether cultivated mangosteen, *G*. *mangostana* var. *malaccensis* and var. *borneensis*, are genetically differentiated, or whether differences between them in morphological traits are due to phenotypic plasticity. Genome (Abu Bakar et al., 2016) and plastome (Wee et al., 2022) datasets of cultivated mangosteen are now available for reference for Single Nucleotide Polymorphism (SNP) calling. Analysis of both nrDNA and cpDNA markers in a large number of samples from across the region would enable inferences about several open questions: (i) patterns of genetic variation in mangosteen and its wild relatives; (ii) whether gene flow occurs between them, and whether any gene flow detected is unidirectional (from wild pollen donors to mangosteen), as expected from results of crossing experiments (Hambali & Natawijaya, 2016); (iii) whether mangosteen originated from numerous initial progenitors that arose from independent events of sexual reproduction. Careful studies of patterns of allelic richness and allele size variation in an adequate number of co‐dominant microsatellite loci would shed light on the contribution of this source of genetic variation relative to somaclonal evolution under asexual propagation of mangosteen. Such studies would also allow testing the expectation from theory that genetic diversity should be greater in mangosteen's wild predominantly sexually reproducing relative (*G*. *mangostana* var. *malaccensis*) than in cultivated mangosteen.

Information about evolutionary ecology, and notably on the breeding system of the wild relatives in their natural habitat, is sorely lacking. Empirical studies on cytogenetics, controlled pollination, pollen viability, phenotypic differences among ploidy series, experimental crosses between mangosteen and various wild relatives, embryo development, and seed germination could provide direct or inferential evidence on the occurrence of polyploidy and apomixis in wild relatives, and their genetic compatibility with mangosteen.

## AUTHOR CONTRIBUTIONS

**Tze Leong Yao:** Conceptualization (equal); visualization (equal); writing – original draft (lead); writing – review and editing (equal). **Mohd Nazre:** Conceptualization (equal); supervision (equal); validation (equal); writing – review and editing (equal). **Doyle McKey:** Conceptualization (equal); validation (equal); writing – original draft (equal); writing – review and editing (equal). **Riina Jalonen:** Conceptualization (equal); validation (equal); writing – review and editing (equal). **Jérôme Duminil:** Conceptualization (equal); funding acquisition (lead); project administration (lead); resources (lead); supervision (equal); validation (equal); writing – review and editing (equal).

## FUNDING INFORMATION

This study was conducted under a studentship co‐funded by (i) Labex AGRO 2011‐LABX‐002 (under I‐Site MUSE framework) coordinated by Agropolis Fondation; (ii) Universiti Putra Malaysia (UPM); and (iii) Southeast Asian Regional Center for Graduate Study and Research in Agriculture (SEARCA). The first author was granted a full salary study leave by Forest Research Institute Malaysia (FRIM). A Dan Nicolson Grant from the International Association for Plant Taxonomy (IAPT) supported field trips and herbarium visits.

## CONFLICT OF INTEREST STATEMENT

We declare no potential competing interest in our study and published findings.

## Data Availability

No new data were created or analyzed in this review; data sharing is not applicable to this article.
